# Artificial Crown and Bridge-Work

**Published:** 1890-02

**Authors:** 


### Bibliographical.

ARTIFICIAL CROWN AND BRIDGE WORK.—By George Evans. Second edition, revised and enlarged, with 547 illustrations. Philadelphia: S. S. White Dental Manufacturing Co., Publishers.

We can justly repeat what we said concerning the first edition of this admirable treatise on a subject that has engaged the attention of the dental practitioner for several years past. We can also add that the present edition is not only a practical and comprehensive treatise on crown-and-bridge-work, but possesses the merit of embracing a careful selection of methods and appliances which greatly enhance the value of this work, and introduces all the new matter which has appeared since the publication of the first edition.

